# MLL1 inhibits the neurogenic potential of SCAPs by interacting with WDR5 and repressing HES1

**DOI:** 10.1038/s41368-023-00253-0

**Published:** 2023-10-18

**Authors:** Chen Zhang, Weilong Ye, Mengyao Zhao, Lujue Long, Dengsheng Xia, Zhipeng Fan

**Affiliations:** 1 Laboratory of Molecular Signaling and Stem Cells Therapy, Beijing Key Laboratory of Tooth Regeneration and Function Reconstruction, Beijing Stomatological Hospital, School of Stomatology, Capital Medical University, Beijing, China; 2 Department of Dental Emergency, Beijing Stomatological Hospital, School of Stomatology, Capital Medical University, Beijing, China; 3 Beijing Laboratory of Oral Health, Capital Medical University, Beijing, China; 4 Research Unit of Tooth Development and Regeneration, Chinese Academy of Medical Sciences, Beijing, China

**Keywords:** Stem cells, Regeneration, Mesenchymal stem cells

## Abstract

Mesenchymal stem cell (MSC)-based therapy has emerged as a promising treatment for spinal cord injury (SCI), but improving the neurogenic potential of MSCs remains a challenge. Mixed lineage leukemia 1 (MLL1), an H3K4me3 methyltransferases, plays a critical role in regulating lineage-specific gene expression and influences neurogenesis. In this study, we investigated the role and mechanism of MLL1 in the neurogenesis of stem cells from apical papilla (SCAPs). We examined the expression of neural markers, and the nerve repair and regeneration ability of SCAPs using dynamic changes in neuron-like cells, immunofluorescence staining, and a SCI model. We employed a coimmunoprecipitation (Co-IP) assay, real-time RT-PCR, microarray analysis, and chromatin immunoprecipitation (ChIP) assay to investigate the molecular mechanism. The results showed that MLL1 knock-down increased the expression of neural markers, including neurogenic differentiation factor (NeuroD), neural cell adhesion molecule (NCAM), tyrosine hydroxylase (TH), βIII-tubulin and Nestin, and promoted neuron-like cell formation in SCAPs. In vivo, a transplantation experiment showed that depletion of MLL 1 in SCAPs can restore motor function in a rat SCI model. MLL1 can combine with WD repeat domain 5 (WDR5) and WDR5 inhibit the expression of neural markers in SCAPs. MLL1 regulates Hairy and enhancer of split 1 (HES1) expression by directly binds to HES1 promoters via regulating H3K4me3 methylation by interacting with WDR5. Additionally, HES1 enhances the expression of neural markers in SCAPs. Our findings demonstrate that MLL1 inhibits the neurogenic potential of SCAPs by interacting with WDR5 and repressing HES1. These results provide a potential therapeutic target for promoting the recovery of motor function in SCI patients.

## Introduction

SCI is one of the most serious neurological diseases in the world, with a prevalence rate of 8-906 patients per million people.^1^ Although it primarily affects younger individuals, its incidence in older populations has also increased in recent years.^2^ The regeneration potential of spinal axons is severely limited owing to several factors, including the intrinsic growth capacity of neurons, insufficient availability of neurotrophic and nerve growth factors and the propensity for the formation of glial scars. These challenges have been recognized as major contributors to the failure of successful repair and functional recovery following SCI.^3,4^ Current treatments for SCI encompass surgical intervention, physiotherapeutic techniques, and pharmacological approaches. Despite the implementation of these strategies, their therapeutic outcomes have yet to meet prevailing expectations.^5^ Thus, a novel, effective therapeutic method is urgently needed for the treatment of SCI. Recently, stem cell transplantation has become a novel alternative treatment for SCI. Stem cells from apical papilla (SCAPs), derived from the root apex of incompletely developed teeth, can secrete a variety of growth factors, are readily availability and have an excellent proliferation capacity.^6,7^ Furthermore, SCAPs hold promise as a therapeutic candidate for the treatment of SCI, owing to their origin from neural crest cells and expression of specific neural stem cell (NSC) markers. These features suggest that SCAPs may exhibit stronger nerve regeneration and repair ability than bone marrow mesenchymal stem cells (BMSCs).^8,9^ Additionally, SCAPs have been shown to release soluble factors that regulate neurite outgrowth in trigeminal neurons, further supporting their potential as effective seed cells for SCI therapy.^10^ Nonetheless, despite these encouraging findings, there is still ample room for improvement in the efficacy of SCAP-based therapies for SCI treatment.

Epigenetic regulation represents a key mechanism for controlling cellular functions. In particular, MLL1 has emerged as a crucial regulator of neural development, exerting specific control over H3K4me3 levels.^11^ Studies have demonstrated that knockout of Kmt2a/Mll1 promotes neurogenic differentiation while inhibiting glial cell differentiation in zebrafish,^11,12^ highlighting the fundamental importance of MLL1 in neural development. The formation of a glial cell scar presents a physical impediment to axonal growth, thereby hindering the recovery of locomotor function following SCI.^13,14^ Prior studies have established that MLL1 exhibits robust expression in neural tissues under hypoxic conditions.^15^ and is critical for the growth of glioblastoma stem-like cells,^16^ implying a potential involvement of MLL1 in gliogenesis. Furthermore, MLL1 has been identified as a key regulator of neuronal plasticity genes associated with memory formation^17^ and has been implicated in regulating working memory ability in mice.^18^ Despite these findings, the impact of MLL1 on the neurogenic potential of human MSCs has yet to be fully elucidated.

MLL1 is a chromatin modification factor that is conserved and known for maintaining lineage-specific gene expression among stem cell populations.^19^ MLL1 activity is largely dependent on its interaction with WD repeat domain 5 (WDR5), as co-binding with WDR5 significantly enhances its H3K4 methyltransferase activity.^20^ WDR5 is involved in the regulation of numerous physiological functions, including self-renewal ability,^21^ osteoblastic and chondrocyte differentiation,^22^ expression of neural genes,^23,24^ and muscle tissue generation,^25^ indicating its vital role in cellular differentiation processes. MLL1 promotes the H3K4me3-mediated maintenance of spinal cord plasticity through its association with WDR5, and disruption of the MLL1-WDR5 interaction has been shown to alleviate neuropathic allodynia.^26^ Studies examining rat gastrocnemius muscles following nerve transection have demonstrated that upregulation of MLL1 and WDR5 expression is partially associated with denervation.^27^ Despite their valuable roles in neurogenesis, the regulatory mechanisms that promote the neurogenic potential of MSCs remain poorly understood. Therefore, further investigation into the role of MLL1 combined with WDR5 in promoting the neurogenic potential of MSCs is warranted.

In the present investigation, SCAPs were employed to explore the role of MLL1 and WDR5 in neurogenesis. Our findings indicate that knock-down of MLL1 in SCAPs exerts restorative effects on motor function in a rat SCI model. Specifically, our results suggest that MLL1 interacts with WDR5 and acts to impede the neurogenic potential of SCAPs.

## Results

### MLL1 knock-down increases the expression of neural markers in SCAPs

**Fig. 1:**
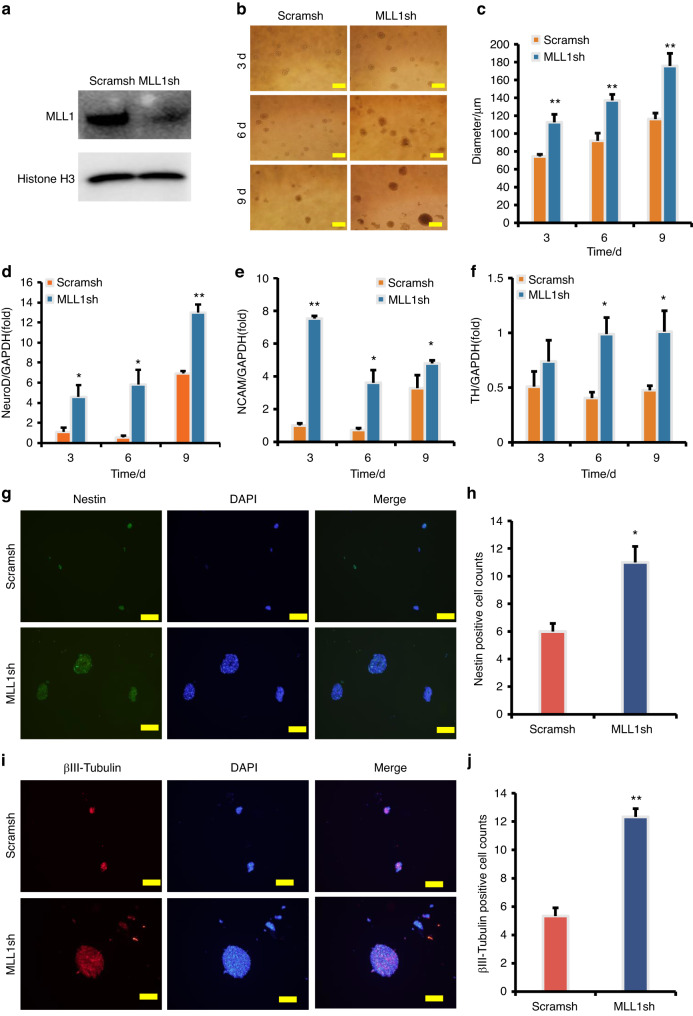
MLL1 knock-down enhanced the expression of neural markers in SCAPs. **a** The MLL1 knock-down efficiency was tested by Western blot. Histone H3 served as an internal control; **b**, **c** MLL1 knock-down increased the neurosphere volume compared to the SCAP/Scramsh group. Scale bar, 100 μm; Real-time RT-PCR analysis revealed that MLL1 knock-down increased the expression of NeuroD (**d**), NCAM (**e**), and TH (**f**) in SCAPs. GAPDH served as an internal control; **g**–**j** The immunofluorescence staining revealed that the numbers of Nestin-positive and βIII-tubulin-positive neurospheres were significantly increased in the SCAP/MLL1sh group compared to the SCAP/Scramsh group. Scale bar, 100 μm. Statistical significance was determined using Student’s t test. All error bars represent SD. (n = 3). *P ≤ 0.05. **P ≤ 0.01

To confirm the identity of the SCAPs, flow cytometry analysis was conducted to detect specific markers. The results indicate positive expression of the surface markers CD90 (99.2%), CD105 (92.8%), and CD146 (88.2%) in SCAPs (Supplementary Fig. 1A–C), while CD34 (0.44%) and CD45 (0.041%) exhibit negative expression in SCAPs (Supplementary Fig. 1D, E). To knock down MLL1 in SCAPs, lentivirus transfection was employed, and the knock-down efficiency was detected by Western blot (Fig. 1a). During neuron induction, we observed morphological changes in SCAPs, indicating their differentiation into neuron-like cells. Specifically, we monitored the dynamic changes of neurosphere at 3, 6, and 9 days and compared these parameters between the SCAP/MLL1sh and the SCAP/Scramsh groups. Our results demonstrated that the SCAP/MLL1sh group exhibited a significant increase in neurosphere volume compared to the SCAP/Scramsh group (Fig. 1b, c). Moreover, we evaluated the expression of neural markers using real-time RT-PCR and found that the expressions of NeuroD and NCAM were higher at 3, 6, and 9 days, and TH expression was higher at 6 and 9 days after induction in the SCAP/MLL1sh group than those in the SCAP/Scramsh group (Fig. 1d–f). Additionally, the immunofluorescence staining and quantification analysis revealed that the numbers of Nestin-positive and βIII-tubulin-positive neurospheres were significantly increased in the SCAP/MLL1sh group compared to the SCAP/Scramsh group (Fig. 1g–j).

### Knock-down of MLL1 in SCAPs can promote functional recovery in a rat SCI model

**Fig. 2:**
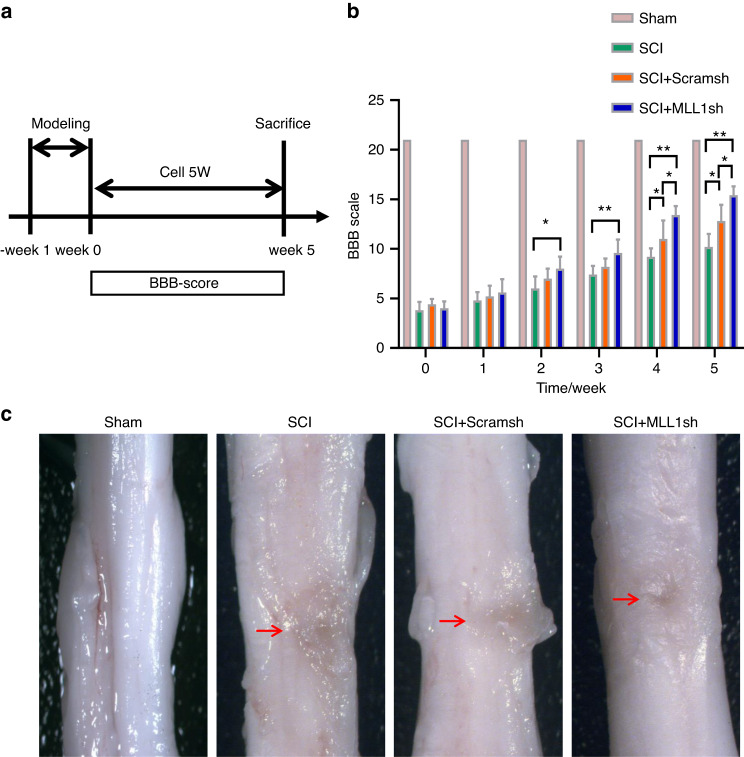
MLL1 knock-downed SCAPs promoted functional recovery after SCI in rat model. **a** Spinal cord function was observed at 1, 2, 3, 4, and 5 weeks using the BBB scale to assess the limb motor function of rats. **b** The BBB scale showed that the SCAP/MLL1sh group gradually exhibited better spinal functional recovery at 4 to 5 weeks compared with that in the SCAP/Scramsh group. **c** Compared with those in the SCI and SCAP/Scramsh groups, the spinal cord morphology of the SCAP/MLL1sh group recovered well. Statistical significance was determined using one-way ANOVA, or the Kruskal–Wallis test. All error bars represent SD. (n = 6). *P ≤ 0.05. **P ≤ 0.01

**Fig. 3:**
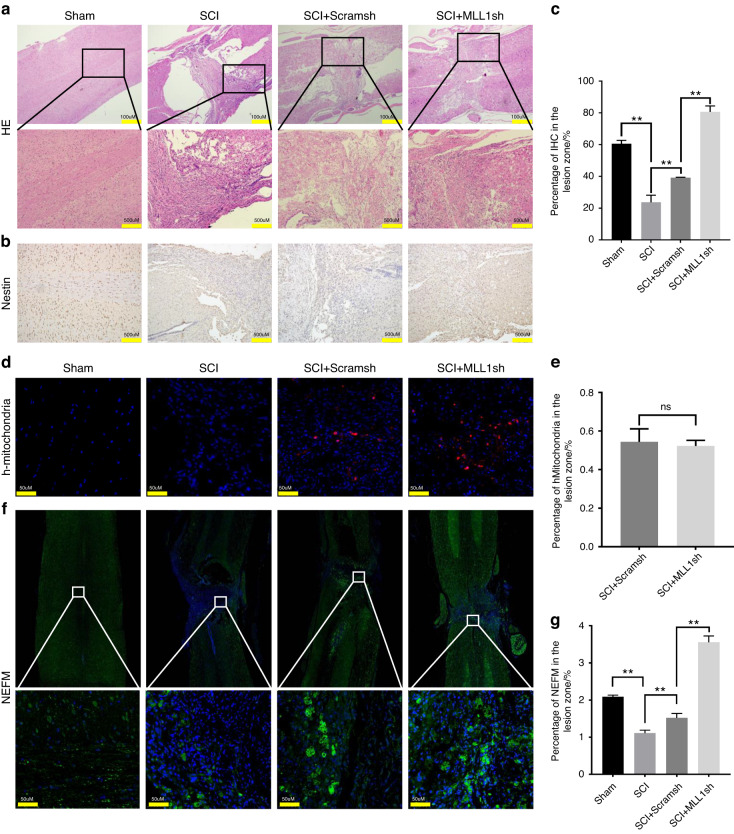
Histopathological changes in the SCI model after SCAP transplantation. **a** HE staining results revealed that the SCAP/MLL1sh group had more vacuolar cells and fewer cavities and scars than the SCI and SCAP/Scramsh groups. Scale bar, 100 μm and 500 μm. **b**, **c** The immunohistochemical results indicated that the SCAP/MLL1sh group exhibited significantly higher levels of Nestin expression compared to the SCAP/Scramsh group. Scale bar, 100 μm. **d**, **e** The immunofluorescence results indicated that h-mitochondria-positive cells were in the injury area in SCAP/Scramsh and SCAP/MLL1sh groups at 5 weeks after transplantation. Scale bar, 50 μm. **f**, **g** Immunofluorescence staining was used to observe the NEFM expression. Scale bar, 50 μm. Statistical significance was determined using one-way ANOVA, or the Kruskal–Wallis test. All error bars represent SD. (n = 6). **P ≤ 0.01

We investigated whether the knock-down of MLL1 in SCAPs could result in better functional recovery in SCI model. To evaluate this possibility, we assessed the motor behavioral deficits of SCI rats using the Basso-Beattie-Bresnahan (BBB) scale at 1, 2, 3, 4, and 5 weeks after cell transplantation (Fig. 2a). Our findings indicate that the rats exhibited significant motor behavioral deficits post-SCI. In the 2nd and 3rd weeks following cell transplantation, there was an improvement in motor function in the SCAP/MLL1sh group compared to the SCI group, and this difference was statistically significant. There was no significant difference observed when compared to the SCAP/Scramsh group. However, in the 4th and 5th weeks, both the SCAP/Scramsh group and the SCAP/MLL1sh group exhibited differences in motor function when compared to the SCI group. Notably, the SCAP/MLL1sh group demonstrated significantly superior motor function recovery compared to the SCAP/Scramsh group, with statistically significant differences observed (Fig. 2b). These subsequent observations suggested a potential improvement in spinal cord function with the transplantation of MLL1 knock-downed SCAPs. Compared with those of the SCI and SCAP/Scramsh groups, the spinal cord morphology in the SCAP/MLL1sh group showed better recovery (Fig. 2c). To assess the effect of SCAP transplantation on SCI-related pathological changes, HE staining was performed to observe the overall damage in spinal cord tissue. Compared to the Sham group, the spinal cord in the SCI group exhibited severe structural damage, characterized by the formation of spinal cord cavities, pathological bleeding, necrosis, and scar formation. On the other hand, The SCAP/Scramsh group showed a reduction in cavities and continuity at the injury site of spinal cord. Furthermore, in comparison to both the SCI and SCAP/Scramsh groups, the SCAP/MLL1sh group exhibited more vacuolar cells and fewer cavities and scars (Fig. 3a). The neural progenitor cells in spinal cord defects was evaluated using immunohistochemical staining of Nestin. A small amount of Nestin expression was observed in the SCI group, with significant difference noted between the SCI and Sham groups. The SCAP/Scramsh group exhibited a higher expression of Nestin than the SCI group, yet it remained significantly lower than that observed in the SCAP/MLL1sh group (Fig. 3b, c). To confirm the presence of implanted SCAPs, a red fluorescent signal indicating human mitochondria-positive cells in the injured spinal cord area was visualized using fluorescence microscopy after SCAP/Scramsh and SCAP/MLL1sh group implantation at 5 weeks (Fig. 3d). Meanwhile, due to the specific fluorescence labeling of transplanted cells with human mitochondria, either the Sham group or the SCI group didn’t exhibit any positive cells (Fig. 3d). The quantification analysis results show that there is no statistically significant difference in the number of human mitochondria-positive cells between the SCAP/Scramsh group and the SCAP/MLL1sh group (Fig. 3e). The expression of NEFM may serve as reliable indicators of SCI repair. Furthermore, immunofluorescence staining was conducted to investigate the changes in NEFM expression. The results revealed a significant decrease in NEFM expression in the SCI group compared to the Sham group. Conversely, the SCAP/Scramsh group exhibited pronounced NEFM expression, which differed significantly from the SCI group. And the NEFM expression significantly increased in the SCAP/MLL1sh group compared to the SCI and the SCAP/Scramsh groups, suggesting that MLL1 knock-down in SCAPs may inhibit axonal degeneration (Fig. 3f, g). Overall, our findings suggest that the transplantation of MLL1 knock-downed SCAPs can accelerate the repair of injured spinal cord tissue and promote motor functional recovery in a rat SCI model

### WDR5 interacted with MLL1, and inhibited the expression of neural markers in SCAPs

**Fig. 4:**
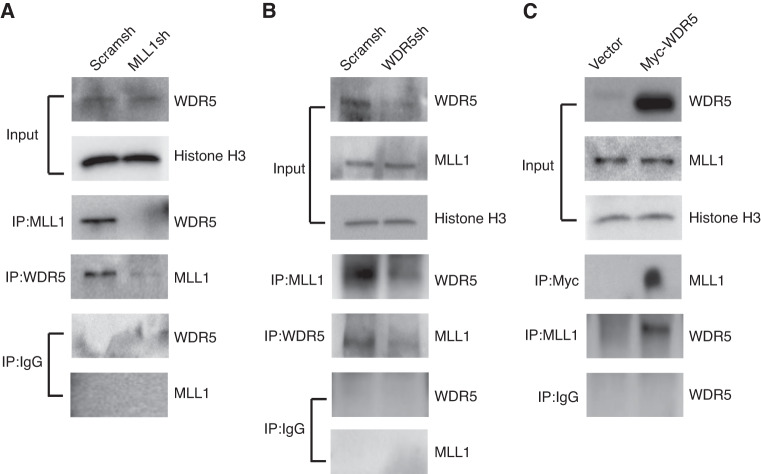
MLL1 binding with WDR5 in SCAPs. **a** MLL1 knock-down reduced the binding of MLL1 and WDR5 in SCAPs; **b** WDR5 knock-down reduced the binding of MLL1 and WDR5 in SCAPs; **c** WDR5 over-expression significantly increased the binding of MLL1 and WDR5 in SCAPs. Histone H3 served as an internal control. IgG was used as the negative control

To identify potential MLL1-binding proteins in SCAPs, we performed a Co-IP assay. Our findings revealed that knock-down of MLL1 in SCAPs led to a reduction in the binding of MLL1 and WDR5 (Fig. 4a). To further validate this observation, WDR5 knock-downed SCAPs were constructed through lentivirus transfection, and the knock-down efficiency was confirmed by Western blot analysis (Fig. 4b). Subsequently, we investigated the effects of WDR5 knock-down on the binding of WDR5 and MLL1 in SCAPs. Our results demonstrated that knock-down of WDR5 led to a decrease in the binding of MLL1 and WDR5, which was consistent with the observations in MLL1 knock-downed SCAPs (Fig. 4b). Next, the Myc-WDR5 sequence was inserted into the retroviral vector, which was used to infect SCAPs. WDR5 over-expression was tested by Western blot (Fig. 4c). Furthermore, WDR5 over-expression significantly increased the binding of MLL1 and WDR5 in SCAPs (Fig. 4c). These findings provide further evidence for the role of WDR5 as a binding partner of MLL1 in SCAPs.

**Fig. 5:**
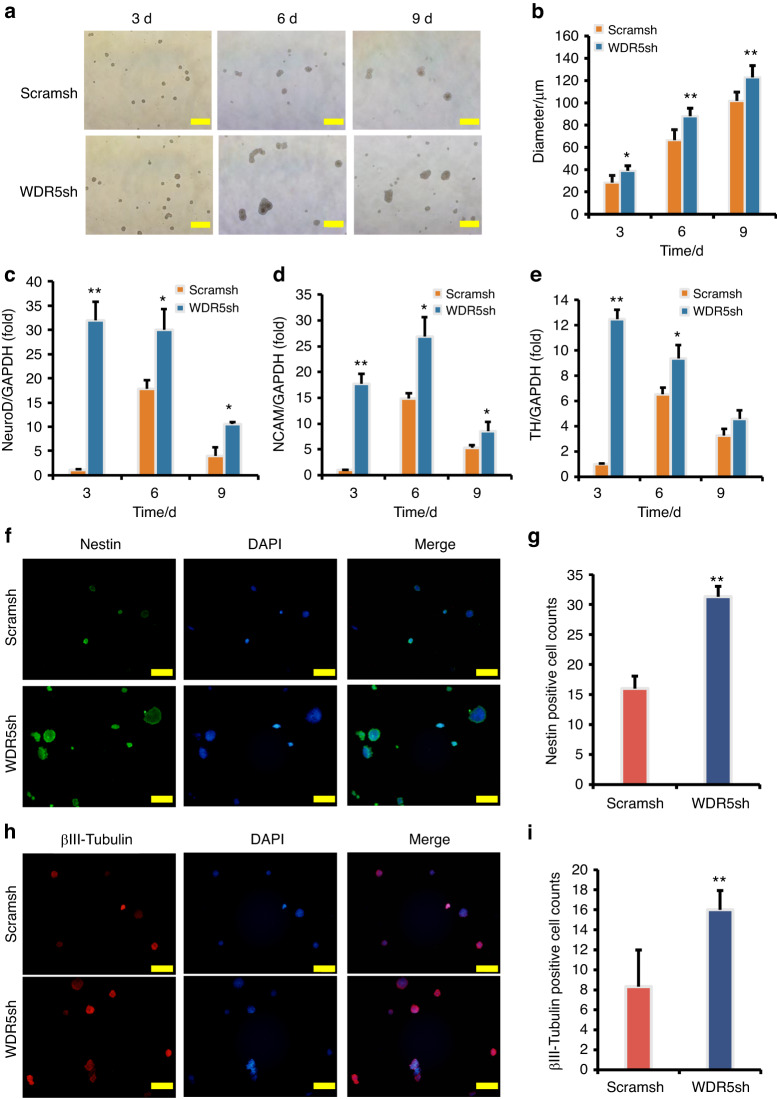
WDR5 knock-down enhanced the expression of neural markers in SCAPs. **a**, **b** WDR5 knock-down increased the neurosphere volume compared to the SCAP/Scramsh group. Real-time RT-PCR analysis revealed that WDR5 knock-down increased the expression of NeuroD (**c**), NCAM (**d**), and TH (**e**) in SCAPs. GAPDH served as an internal control. **f**–**i** The immunofluorescence staining revealed that the numbers of Nestin-positive and βIII-tubulin-positive neurospheres were significantly increased in the SCAP/WDR5sh group compared to the SCAP/Scramsh group. Statistical significance was determined using Student’s t test. All error bars represent SD. (n = 3). *P ≤ 0.05. **P ≤ 0.01

Subsequently, WDR5 was knocked down in SCAPs for functional assays. Morphological changes were observed in neuron-like cells during neuron induction. Notably, the SCAP/WDR5sh group exhibited a significant increase in the neurosphere volume compared to the SCAP/Scramsh group (Fig. 5a, b). Real-time RT-PCR analysis revealed that neural markers such as NeuroD, NCAM, and TH were up-regulated in SCAPs following WDR5 knock-down compared to the SCAP/Scramsh group (Fig. 5c–e). Additionally, the immunofluorescence staining and quantification analysis revealed that the numbers of Nestin-positive and βIII-tubulin-positive neurospheres were significantly increased in the SCAP/WDR5sh group compared to the SCAP/Scramsh group (Fig. 5f–i).

**Fig. 6:**
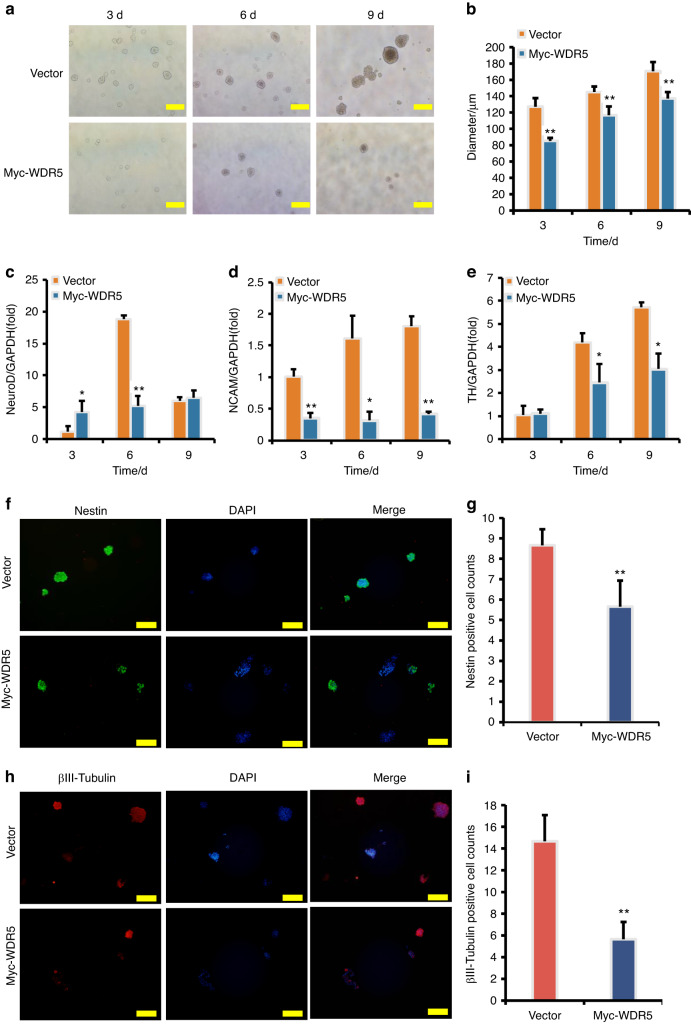
WDR5 overexpression inhibited the expression of neural markers in SCAPs. **a**, **b** WDR5 over-expression decreased the neurosphere volume compared to the Vector group. Real-time RT-PCR analysis revealed that WDR5 over-expression decreased the expression of NeuroD (**c**), NCAM (**d**), and TH (**e**) in SCAPs. GAPDH served as an internal control. **f**–**i** The immunofluorescence staining revealed that the numbers of Nestin-positive and βIII-tubulin-positive neurospheres were significantly decreased in the WDR5 over-expressed SCAPs compared to the SCAP/Vector group. Statistical significance was determined using Student’s t test. All error bars represent SD. (n = 3). *P ≤ 0.05. **P ≤ 0.01

We further investigate the role of WDR5 in regulating the neurogenic potential of SCAPs via over-expression of WDR5. The dynamic changes in neuron-like cells were monitored for 3, 6, and 9 days, revealing that the WDR5 over-expressed group exhibited a significant decrease in neurosphere volume compared to the SCAP/Vector group (Fig. 6a, b). After 9 days of neuron induction, real-time RT-PCR analysis suggested that the expression levels of NeuroD at 3, and 6 days, NCAM at 3, 6, and 9 days, and TH at 6, and 9 days in the WDR5 over-expressed group were significantly decreased compared to those in the SCAP/Vector group (Fig. 6c, e). Consistent with these findings, immunofluorescence staining and quantification analysis revealed that the numbers of Nestin-positive and βIII-tubulin-positive neurospheres were significantly decreased in the WDR5 over-expressed group compared to the SCAP/Vector group (Fig. 6f, i). Together, these results provide further evidence supporting the inhibitory effects of WDR5 over-expression on neurogenesis and suggest a potential therapeutic strategy for promoting neurogenesis by modulating WDR5 levels.

### MLL1 and WDR5 directly regulate HES1 transcription through H3K4me3 methylation

**Fig. 7:**
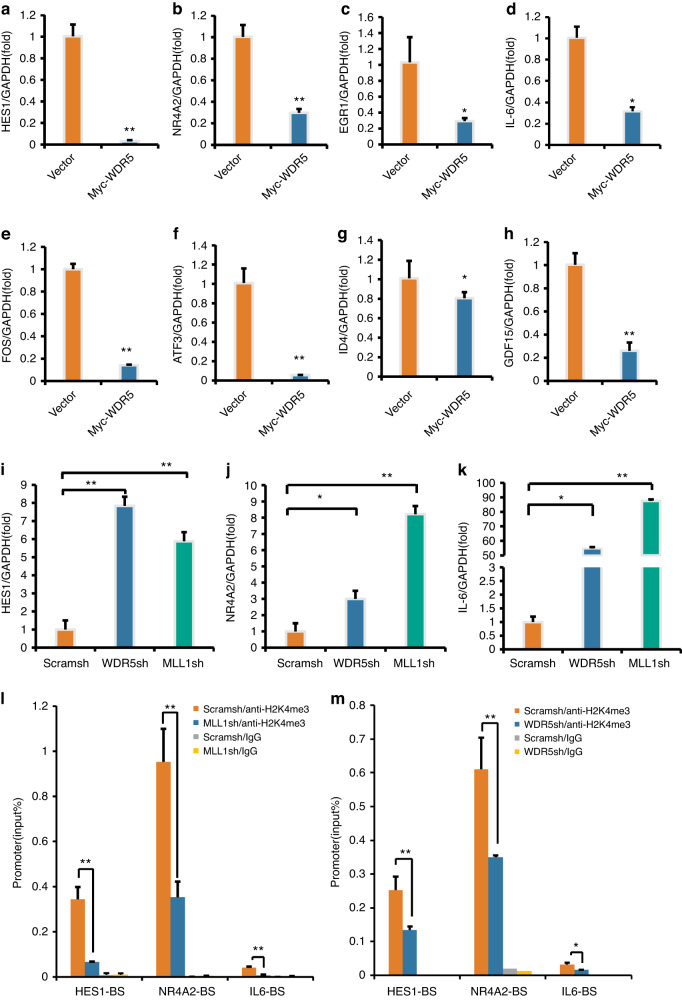
Knocking down MLL1 or WDR5 promotes the expression of HES1, NR4A2, and IL-6 through H3K4me3 methylation. Real-time RT-PCR analysis revealed that the expression of neural-related genes in the SCAP/Myc-WDR5 group was significantly reduced, including HES1 (**a**), NR4A2 (**b**), EGR1 (**c**), IL-6 (**d**), FOS (**e**), ATF3 (**f**), ID4 (**g**), and GDF15 (**h**). GAPDH served as an internal control. **i**–**k** Real-time RT-PCR analysis revealed that the knock-down of WDR5 or MLL1 up-regulated HES1, NR4A2, and IL-6 expression in SCAPs. GAPDH served as an internal control. **l**–**m** The ChIP assay results revealed that depletion of MLL1 or WDR5 decreased the enrichment of H3K4me3 in the HES1, NR4A2, and IL-6 promoters. Statistical significance was determined using one-way ANOVA, or the Kruskal–Wallis test. All error bars represent SD. (n = 3). *P < 0.05. **P ≤ 0.01

To further investigate the co-regulatory role of MLL1 and WDR5 in regulating the neurogenic potential of SCAPs, we performed microarray analysis to identify potential target genes associated with this process. Our findings revealed 35 differentially expressed genes in the WDR5 over-expressed group compared to the Vector group, with 9 genes up-regulated and 26 genes down-regulated in SCAPs over-expressing WDR5 (Supplementary Table 2). The up-regulated genes included CYP1B1, PRSS3, and NPTX1, while the down-regulated genes included HES1, NR4A2, EGR1, IL-6, FOS, ATF3, ID4, and GDF15. To further validate these findings, we performed real-time RT-PCR to detect the expression levels of differentially expressed genes after over-expression of WDR5. Our results showed that, compared to the Vector group, the expression of neuro-related genes such as HES1, NR4A2, EGR1, IL-6, FOS, ATF3, ID4, and GDF15 in the WDR5 over-expressed SCAP were significantly reduced (Fig. 7a–h). Further investigation of the co-regulatory role of MLL1 and WDR5 in the regulation of gene expression was performed. Our results indicated that the knock-down of WDR5 or MLL1 led to the up-regulation of HES1, NR4A2, and IL-6 expression in SCAPs (Fig. 7i–k). Our experimental results also demonstrate that knocking down of MLL1 in SCAPs results in a reduction in H3K4me3 level (Supplementary Fig. 2). Furthermore, we confirmed that MLL1 depletion decreased the level of H3K4me3 in the HES1, NR4A2, and IL-6 promoter regions (Fig. 7L). The enrichment of H3K4me3 in the HES1, NR4A2, and IL-6 promoter regions was also significantly reduced in the SCAP/WDR5sh group compared to the SCAP/Scramsh group (Fig. 7m).

**Fig. 8:**
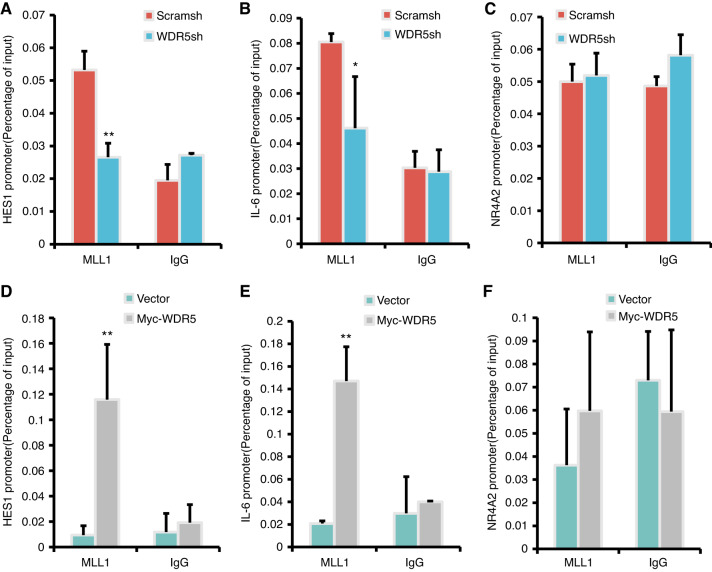
MLL1 directly regulated the transcription of HES1 and IL-6 by binding with WDR5. The ChIP assay results showed that WDR5 knock-down reduced the binding of MLL1 to the HES1 (**a**) and IL-6 promoters (**b**), except for the NR4A2 promoter region (**c**); The ChIP assay results showed that WDR5 over-expression increased the binding of MLL1 to the HES1 (**d**) and IL-6 promoters (**e**), except for the NR4A2 promoter region (**f**). Statistical significance was determined using Student’s t test. All error bars represent SD. (n = 3). *P ≤ 0.05. **P ≤ 0.01

To demonstrate the regulatory effect of the MLL1-WDR5 protein complex on downstream target genes such as HES1, IL-6, and NR4A2, we performed ChIP assays. Our results indicated that WDR5 knock-down led to a decrease binding of MLL1 into the HES1 and IL-6 promoters compared to that in the SCAP/Scramsh group (Fig. 8a, b). However, binding of MLL1 was not enriched in the NR4A2 promoter region (Fig. 8c). To further confirm the regulatory role of MLL1 and WDR5 on HES1, IL-6, and NR4A2, we conducted ChIP assays in the WDR5 over-expressed SCAPs. Our findings revealed that compared to the SCAP/Vector group, WDR5 over-expression increased MLL1 enrichment in the HES1 and IL-6 promoter regions (Fig. 8d, e), while the MLL1 enrichment in NR4A2 promoter region was not affected (Fig. 8f). Together, these results provide further evidence supporting the co-regulatory role of MLL1 and WDR5 in the regulation of HES1 and IL-6.

### HES1 promoted the expression of neural markers in SCAPs

**Fig. 9:**
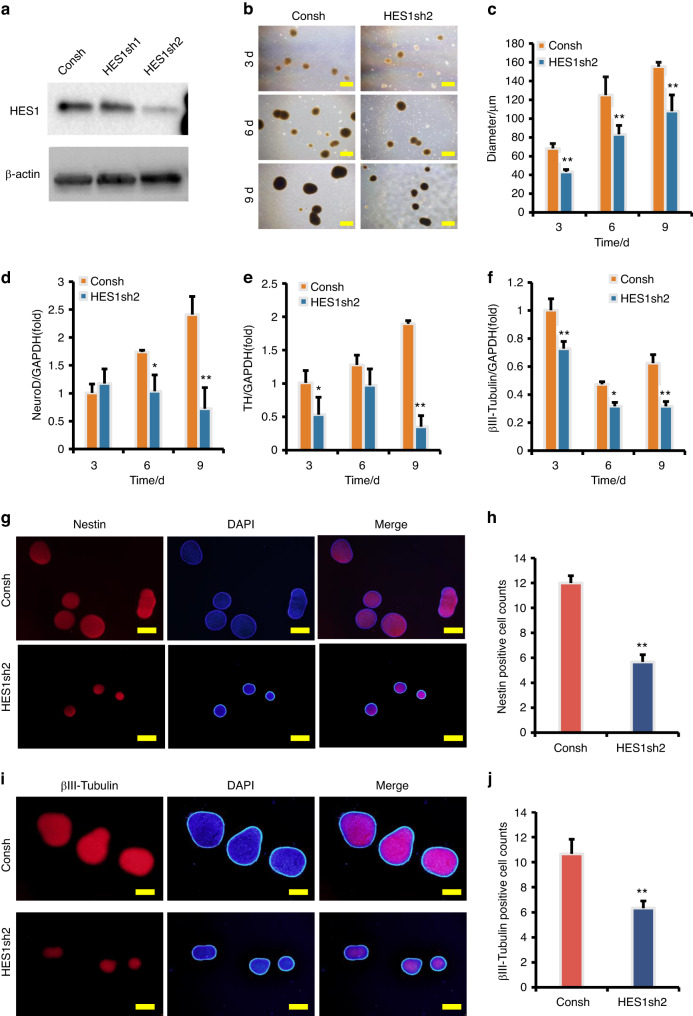
HES1 knock-down reduced the expression of neural markers in SCAPs. **a** The HES1 knock-down efficiency was tested by Western blot. β-actin served as an internal control; **b**, **c** HES1 knock-down reduced the neurosphere volume compared to the SCAP/Consh group; Real-time RT-PCR analysis revealed that HES1 knock-down reduced the expression of NeuroD (**d**), TH (**e**), and βIII-tubulin (**f**) in SCAPs. GAPDH served as an internal control. **g**–**j** The immunofluorescence staining revealed that the numbers of Nestin-positive and βIII-tubulin-positive neurospheres were significantly decreased in the SCAP/HES1sh group compared to the SCAP/Consh group. Statistical significance was determined using Student’s t test. All error bars represent SD. (n = 3). *P ≤ 0.05. **P ≤ 0.01

HES1 was knocked down in SCAPs for functional assays, and the knock-down efficiency was detected by Western blot (Fig. 9a). The results indicate that, compared to the SCAP/Consh group, the expression of HES1 was significantly reduced in the SCAP/HES1sh2 group. Then the SCAP/HES1sh2 group exhibited a significant reduction in neurosphere volume compared to the SCAP/Consh group (Fig. 9b, c). Real-time RT-PCR analysis revealed down-regulation of neural markers such as NeuroD, TH, and βIII-tubulin after HES1 knock-down in SCAPs compared to the SCAP/Consh group (Fig. 9d–f). Consistent with these findings, immunofluorescence staining and quantification analysis revealed that the numbers of Nestin-positive and βIII-tubulin-positive neurospheres were significantly decreased in the SCAP/HES1sh2 group compared to the SCAP/Consh group (Fig. 9g–j).

**Fig. 10:**
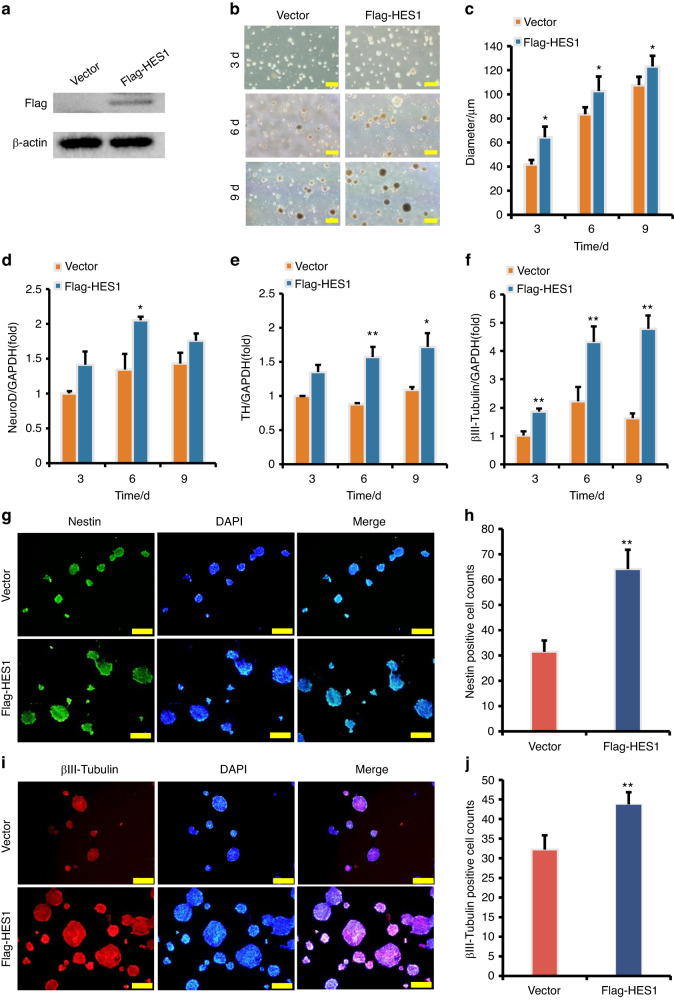
HES1 over-expression increased the expression of neural markers in SCAPs. **a** The HES1 over-expression efficiency was tested by Western blot. β-Actin served as an internal control; **b**, **c** HES1 over-expression increased the neurosphere volume compared to the SCAP/Vector group; **d**–**f** Real-time RT-PCR analysis revealed that HES1 over-expression increased the expression of NeuroD (**d**), TH (**e**), and βIII-tubulin (**f**) in SCAPs. GAPDH served as an internal control. **g**–**j** The immunofluorescence staining revealed that the numbers of Nestin-positive and βIII-tubulin-positive neurospheres were significantly increased in the HES1 over-expressed SCAPs compared to the SCAP/Vector group. Statistical significance was determined using Student’s t test. All error bars represent SD. (n = 3). *P ≤ 0.05. **P ≤ 0.01

To further evaluate the function of HES1, the Flag-HES1 sequence was inserted into the retroviral vector, which was used to infect SCAPs. Western blot analysis was performed to confirm the HES1 over-expression efficiency (Fig. 10a). We monitored the dynamic changes in neuron-like cells for 3, 6, and 9 days, revealing that the HES1 over-expressed SCAPs exhibited a significant increase in neurosphere volume compared to the Vector group (Fig. 10b, c). Real-time RT-PCR analysis indicated that the expression levels of NeuroD, TH, and βIII-tubulin were significantly higher in the SCAP/Flag-HES1 group than those in the SCAP/Vector group after induction (Fig. 10d–f). Furthermore, immunofluorescence staining and quantification analysis revealed that the numbers of Nestin-positive and βIII-tubulin-positive neurospheres were significantly increased in the HES1 over-expressed SCAPs compared to the SCAP/Vector group (Fig. 10g–j).

## Discussion

Neurological recovery following SCI has been explored through the use of MSCs in animal experiments and clinical studies. The primary cause of motor dysfunction after SCI is a disruption in the functional connection of the central nervous system, leading to the formation of a cavity at the injury site and subsequent scar tissue formation. MSC transplantation has been shown to promote axonal regeneration^28^ and decrease the secretion of proinflammatory cytokines.^29^ However, the control of glial scar formation and axon regeneration remains a significant challenge to be addressed. Considering the potential limitations of growth factors, such as their potential side effects, transient expression, and limited therapeutic efficacy when systemically administered, gene-modified stem cells represent a promising alternative for addressing these deficiencies in the context of neural injury repair.^30^ Notably, gene-modified stem cells can provide more precise and controlled regulation of neural differentiation, potentially leading to a more balanced and controlled neurogenic response. It’s important to acknowledge that while promising, gene-modified stem cell therapy is still an evolving field, and further research is required to fully elucidate its advantages and limitations compared to conventional growth factor-based approaches.

SCAPs derived from cranial neural crest cells have been identified as a promising treatment for central nervous system injury. Studies have demonstrated that SCAPs can differentiate into nerve cells and offer neuroprotection while simultaneously reducing glial cell generation and promoting functional recovery following SCI.^31^ Additionally, recent research has shown that genetically modified MSCs can be used to increase intrinsic axon growth and reduce repressive environments for axon regeneration.

Notably, our in vitro studies have revealed that the knock-down of MLL1 can increase the volume of neurospheres formed by SCAPs, leading to the formation of unique MSC-derived neurospheres that possess characteristics of both MSCs and NSCs.^32^ These findings suggest that modified SCAPs hold promise as an effective treatment for neurological injuries. Furthermore, our investigation revealed that the knock-down of MLL1 during short neural induction increases the expression of neural markers such as NeuroD, NCAM, and TH. NeuroD is vital for maintaining cellular maturation and status^33^ and is not expressed in glial tumor cells.^34,35^ NCAM and TH play a crucial role in neurogenesis, differentiation, and survival, with their loss of function leading to nerve regeneration disorders.^36–38^ The up-regulation of these indicators suggests that MLL1 knock-down may impede SCAP differentiation into astrocytes and promote neurogenesis. Subsequently, our study aimed to demonstrate the therapeutic potential of MLL1 knock-down SCAPs for SCI treatment. After 5 weeks of cell transplantation, the MLL1 knock-down SCAPs transplant exhibited motor functional recovery up to approximately 75% of the preinjury level. Additionally, the MLL1 knock-down SCAPs transplant displayed fewer cavities and less scar tissue, along with more Nestin-positive cells at the injury site in SCI model. Furthermore, the expression of Nestin, an early neural marker.^39^ confirmed the repair of spinal cord tissue after SCAP transplantation. Importantly, viable SCAPs were observed in the cells transplant groups, indicating the involvement of SCAPs in repair of spinal cord injury. Even more surprisingly, compared to both SCI and the Scramsh groups, the MLL1 knock-down SCAPs transplant exhibited a higher detection of NEFM-positive cells at the interface between neural tissue and transplanted SCAPs. NEFM is a crucial neurofilament protein expressed in axons and neuron cell bodies, and its dysregulation can result in axonal cytoskeletal defects, thereby indirectly reflecting the extent of axonal damage and repair after SCI.^40,41^ This evidence confirms that the knock-down of MLL1 accelerated the in vivo neurogenesis of SCAPs. To further investigate the regulatory role of MLL1 in the neurogenic potential of SCAPs, we examined its co-binding proteins. Our study revealed that MLL1 binds to WDR5 to form protein complex in SCAPs.

Moreover, we performed microarray analysis to identify potential target genes associated with this process. Our findings revealed that over-expression of WDR5 resulted in HES1, NR4A2, EGR1, IL-6, FOS, ATF3, ID4, and GDF15 down-regulated in SCAPs. Further analysis revealed that the knock-down of either MLL1 or WDR5 increased the expression of HES1, IL-6, and NR4A2. Notably, loss of Notch signaling is known to result in premature neurogenic differentiation and gliosis defects during nervous system development,^42,43^ which is highly similar to the phenotype observed in kmt2a (mll1)-defective embryos. The HES1 is a downstream target of the Notch signaling pathway, playing a promoting role in the differentiation and proliferation of neural stem cells.^44^ Elevated levels of IL-6 is present in the nervous system and can trigger cellular responses, mediate inflammation, play a role in neurogenesis, contribute to the development of neural tumors, influence cell growth and survival, and co-localize with neurotrophic factors. Additionally, elevated levels of IL-6 expression in the hippocampus of both newborn and adult have been shown to promote the differentiation of hippocampal neural progenitor cells into neurons.^45,46^ NR4A2 is a transcription factor that plays a role in the differentiation of neural stem cells (NSCs) into dopaminergic neurons and is involved in neurogenesis.^47^ Abnormal expression of NR4A2 can result in impaired hippocampal neurogenesis.^48^ NR4A2 also functions to inhibit the activation of inflammatory genes by NF-kB in microglia.^49^ These evidences suggested that the alterations in these target genes regulated by MLL1 may modulate the proliferation, survival, and immune response of SCAP, indicated the potential effect of MLL1 on these functions of SCAPs. Then the ChIP results indicate that the knock-down of MLL1 or WDR5 results in a reduction in H3K4me3 levels at the promoter regions of HES1, IL-6, and NR4A2. This impairment in H3K4me3 enrichment maybe attributed to the regulation effect of MLL1 and WDR5 on these target genes.^26^ Further analysis demonstrated that WDR5 can influence the enrichment of MLL1 in the promoter regions of HES1 and IL-6, not on NR4A2 promoter. This suggests that MLL1 and WDR5 protein complex may regulate HES1 and IL-6 expression through H3K4me3 methylation, while NR4A2 may be regulated by other mechanisms.

Previous studies have highlighted the essential role of HES1 in NSC proliferation.^50^ Co-culture of human NSCs with human MSCs and upregulation of Notch-1 and HES1 expression significantly increased the NSC proliferation ability and promoted their differentiation potential, indicating that Notch-HES1 signaling may mediate mesodermal-MSC ectodermal neurogenesis.^44,51,52^ Reports on the neural trans-differentiation of MSCs have revealed that MSCs expressing neuron-specific proteins migrate widely and differentiate into neurons to alleviate neuronal dysfunction.^53,54^ In summary, the selection of HES1 as the focal point in neural research is based on its established role in NSC proliferation, its involvement in the Notch-HES1 signaling pathway, and its potential relevance in neural trans-differentiation processes, as opposed to IL-6. So we selected HES1 as target gene for further investigation. Our research confirmed that over-expression of HES1 enhanced the expression of neural markers in SCAPs, while knock-down of HES1 suppressed the expression of these neural markers in SCAPs. These findings provided a promising therapeutic target of HES1 for improving the efficacy of SCAP-based treatments for SCI.

In summary, our study identified MLL1 and WDR5 as inhibitory regulators of the neurogenic potential of SCAPs. HES1, a downstream target gene of MLL1 and WDR5, was found to positively regulate the neurogenic potential of SCAPs. Mechanistically, we propose that WDR5 interacts with its binding partner, MLL1, to modulate HES1 transcription by assisting MLL1 recruitment to the HES1 promoter region and modifying the H3K4me3 methylation. Our findings provide new insights into the potential mechanisms that promote the neurogenic potential of dental-MSCs and elucidated the potential therapeutic targets for promoting functional recovery after SCI.

## Methods

### Cell culture and characterization

SCAPs were purchased from Shanghai Anwei Biotechnology Co., Ltd. (Shanghai, China). The cultures of SCAPs were described in our previous work.^55^ SCAPs were cultured as previously described to characterize the cells, they were aliquoted into sterile tubes, and then, anti-human antibodies including CD90 (cat no. ab225; Abcam), CD105 (cat no. ab11414; Abcam), CD146 (cat no. ab75769; Abcam), CD34 (cat no. ab81289; Abcam), and CD45 (cat no. ab10558; Abcam) were added at a 1:500 dilution to each tube. The tubes were incubated at 4 °C for 1 h in the dark. Following three washes with 2% fetal bovine serum (FBS) in PBS, the cells were further incubated with secondary antibodies in the dark for 1 h. Subsequently, flow cytometry was employed to detect cell surface markers.

### Plasmid construction and viral infection

The plasmids used in this experiment were constructed according to standard methods. The Myc-tag combined with the full-length sequence of WDR5 was inserted into the pQCXIN retroviral vector. The Flag-tag combined with the full-length sequence of HES1 was inserted into the pQCXIN retroviral vector. Short hairpin RNAs (shRNAs) of WDR5 and MLL1 were inserted into the pLKO.1 lentiviral vector. Subsequently, virus packaging strictly followed the manufacturer’s protocol (Clontech Laboratories or Addgene). The target sequences for the shRNAs were as follows: MLL1 shRNA (MLL1sh) 5’-AAGTGGAATGTTTAACAGTGC-3’; WDR5 shRNA (WDR5sh) 5’- GCCAAACTATGCTCTAAAG -3’. The Scramble shRNA (Scramsh) was purchased from Addgene. HES1 shRNA (HES1sh) 5’-GCTAAGGTGTTTGGAGGCTTC-3’. The Control shRNA (Consh) 5’-TTCTCCGAACGTGTCACGTTTC-3’.

### Neurogenic differentiation and immunofluorescence staining

To induce SCAP neurogenesis, we seeded 1×10^6^ cells into each ultralow adsorption dish (Corning, USA) and cultured them for 9 days. The neuron induction medium was described in our previous work.^56^ Half of the neuron induction medium was changed every 3 days. After neuron induction, dynamic changes in neuron-like cells were observed for 3 days, 6 days, and 9 days under a microscope. After 9 days of neuron induction, immunofluorescence staining was applied to detect the markers of neurogenesis as described in our previous work.^56^ The primer antibodies were as follows: Nestin (Cat No. ab 6320, Abcam, UK), βIII-tubulin (Cat No. T3952, Sigma, USA). At the conclusion of the experiment (5 weeks post-SCI), animals were euthanized by transcardiac perfusion with 4% paraformaldehyde in 0.9% sodium chloride. The spinal cord tissues, which had been previously embedded, were then sectioned into 5 μm slices and subjected to immunofluorescence staining. Immunofluorescence staining was performed according to a previous study.^28^ The primer antibodies were as follows: Nestin (Cat No. ab 6320, Abcam, UK), βIII-tubulin (Cat No. T3952, Sigma, USA), NEFM (Cat No. OMA1-06110, Invitrogen, USA), and h-mitochondria (Cat No. ab 92824, Abcam, UK).

### Coimmunoprecipitation (Co-IP) assay

IP lysis buffer combined with the protease inhibitor cocktail was applied to lyse the SCAPs. The collected protein was rotated overnight with primary antibody at 4 °C, and then 30 μL of protein A/G agarose (Sigma) was added for 2 h. The collected immunoprecipitation complexes were washed with cold IP buffer 3 times. Immunoprecipitated proteins were used for Western blot analysis. The primary antibodies were as follows: WDR5 (Cat No. sc-393080, Santa Cruz, USA), MLL1 (Cat No. 14197 S, Cell Signaling Technology, USA) and Histone H3 (Cat No. SC-56616, Santa Cruz, USA). The detection of the MYC-tag label was carried out using a commercial assay kit (Cat No. 23620, Thermo Scientific^TM^, USA) purchased from Thermo Fisher Company.

### Western blot analysis

Protein extraction and gel electrophoresis were performed as described in our previous work.^57^ The primary antibodies were as follows: WDR5 (Cat No. sc-393080, Santa Cruz, USA), MLL1 (Cat No. 14197 S, Cell Signaling Technology, USA), Flag (Cat No. 66008-4-Ig, Proteintech, USA), HES1 (Cat No. ab 108937, Abcam, UK), H3K4me3 (Cat No. 9751 s, Cell Signaling Technology, USA), Histone H3 (Cat No. SC-56616, Santa Cruz, USA) and β-actin (Cat No. C1313, Applygen, China).

### Real-time reverse transcriptase-polymerase chain reaction (real-time RT-PCR)

The total RNA of the SCAPs was extracted with an extraction kit (RC112-01, Vazyme, China). One microgram of RNA was used to reverse transcribe into cDNA according to the standard procedure of the kit (Invitrogen). Real-time PCRs were performed as described in our previous work.^58^ Glyceraldehyde 3-phosphate dehydrogenase (GAPDH) was used as an internal reference gene to analyze the relative mRNA level. The primers used in this study are shown in Supplementary Table 1.

### Spinal cord injury model

Female Sprague-Dawley rats aged 6-8 weeks were used for the SCI model and were purchased from Vital River Laboratory Animal Technology Co., Ltd. Rats were anesthetized with sodium pentobarbital (40 mg/kg, ip). The spine was exposed between T9 and T10, and spinal cord hemisection at T10 was performed according to a previous method.^59^ A 5-μL cell suspension containing 2×10^6^ cells were added to a 30-µL ECM gel (Cat No. E1270, Sigma, USA) and implanted at the site of the SCI. Sprague-Dawley rats were randomly divided into the sham, SCI, SCI+Scramsh, and SCI+MLL1sh groups (*n* = 6). The animal experiments were approved by the Ethics Committee of the Capital Medical University Affiliated with Beijing Stomatological Hospital (ethics approval number: KQYY-202112-005).

### Behavioral testing

We used the BBB scale to investigate the exercise ability of rats.^60^ Each rat was evaluated by two experimenters who were blinded to the treatment group. Limb movement, gait, coordination, and paw placement were evaluated at 1 week, 2 weeks, 3 weeks, 4 weeks, and 5 weeks after injury.

### Histological analyses

The rats were sacrificed at 5 weeks following SCAP transplantation, and then the spinal cords containing the injured site were collected. The spinal cord was removed and postfixed for 24 h with 4% paraformaldehyde and 30% sucrose solution at 4 °C. Tissues were cut at 5-µm thickness and stained using hematoxylin‐eosin (HE). The expression of Nestin in the spinal cord was detected by immunohistochemistry using a Nestin antibody (Cat No. ab 6320, Abcam, UK).

### Microarray analysis

The PrimeView™ Human Gene Expression Array was used for gene expression profiling. Following the methods of previous studies.^61^ Affymetrix GeneChip Operating Software was used to analyze the differentially expressed genes. The selected threshold set for differentially expressed mRNAs was a fold change≥2 and a p value ≤ 0.05.

### Chromatin immunoprecipitation (ChIP) assays

The ChIP assay was performed using a ChIP assay detection kit (Merck Millipore, Darmstadt, Germany), as previously described.^58^ DNA precipitation was performed using an anti-H3K4me3 antibody (Abcam, Cambridge, U.K.). The quantification results are shown as the percentage of input DNA. The primers targeting the target gene promoter were as follows: HES1 promoter binding site (HES1-BS), forward 5’-ATGAAAAGGGAAAGGGTGGT-3’ and reverse 5’-GGGTGGAAGGAAACGGTATT-3’; NR4A2 promoter binding site (NR4A2-BS), forward 5’-GGGCGGTTTTATTGTTTCCT-3’ and reverse 5’-CCCGCGCTATAAATAACCTG-3’; IL-6 promoter binding site (IL-6-BS), forward 5’-CGGTGAAGAATGGATGACCT-3’ and reverse 5’- GTGACCTCTGTTGGGCATTT -3’. The H3K4me3 (Cat No. 9751 s, Cell Signaling Technology, USA), MLL1 (Cat No. 14197 S, Cell Signaling Technology, USA), and IgG (Cat No. 7076 S, Cell Signaling Technology, USA) antibodies were used.

### Statistical analyses

Each experiment was repeated at least 3 times. GraphPad 5 (GraphPad Software, La Jolla, CA, USA) or SPSS 17 (IBM Corporation, New York, NY, USA) was used for statistical calculations. Statistical analysis was performed using Student’s t test, one-way ANOVA, or the Kruskal–Wallis test depending on the characteristics of the dataset and its distribution. For all analyses, p ≤ 0.05 was regarded as statistically significant.

### Supplementary information

Supplementary Table 1. Primers sequences used in the Real-time RT-PCR

Supplementary Table 2. The differentially expressed genes in WDR5 overexpressed SCAP compared with Vector group

Supplementary Figure Legends

Supplementary Figure 1

Supplementary Figure 2
