# Supplementary material for: MLL1 inhibits the neurogenic potential of SCAPs by interacting with WDR5 and repressing HES1
Source: Int J Oral Sci. 2023 Oct 18;15:48. doi: 10.1038/s41368-023-00253-0 (PMC10584904; doi:10.1038/s41368-023-00253-0)
Supplement: Supplementary file 1 — Supplementary Table 1. Primers sequences used in the Real-time RT-PCR [file 41368_2023_253_MOESM1_ESM.doc]

**Supplementary Table 1. Primers sequences used in the Real-time RT-PCR**

Gene Symbol Primer Sequences (5’-3’)

| GAPDH-F | CGGACCAATACGACCAAATCCG |
| --- | --- |
| GAPDH-R | AGCCACATCGCTCAGACACC |
| NeuroD-F | CGACTGACCCCTACTCCTACCAGTCG |
| NeuroD-R | TGGAAGACATGGGAGCTGTCC |
| NCAM-F | CGGGACCTGGAGGACTTCTACCCG |
| NCAM-R | ACCATGTGCCCATCCAGAGTC |
| TH-F | CCGAGCTGTGAAGGTGTTTGA |
| TH-R | CGGGCCGGGTCTCTAGAT |
| HES1-F | CCACCCCTCCTCCTAAACTC |
| HES1-R | TCCTCTTCTCTCCCAGTATTCA |
| NR4A2-F | TGTTGGGATGGTCAAAGAAG |
| NR4A2-R | TTGGACCTGTATGCTAATCG |
| EGR1-F | CCACCACGTACTCCTCTGTT |
| EGR1-R | GGTTGCTGTCATGTCCGAAA |
| IL-6-F | ACTCACCTCTTCAGAACGAATTG |
| IL-6-R | CCATCTTTGGAAGGTTCAGGTTG |
| FOS-F | TTACTACCACTCACCCGCAG |
| FOS-R | AGTGACCGTGGGAATGAAGT |
| ATF3-F | GGTTAGGACTCTCCACTCAA |
| ATF3-R | AGACAGTAGCCAGCGTCCTT |
| ID4-F | CTGTGCCTGCAGTGCGATAT |
| ID4-R | ACTTTCTTGTTGGGCGGGAT |
| GDF15-F | GACCCTCAGAGTTGCACTCC |
| GDF15-R | GCCTGGTTAGCAGGTCCTC |
| βIII-Tubulin-F | GGCCAAGGGTCACTACACG |
| βIII-Tubulin-R | GCAGTCGCAGTTTTCACACTC |
