# Supplementary material for: MLL1 inhibits the neurogenic potential of SCAPs by interacting with WDR5 and repressing HES1
Source: Int J Oral Sci. 2023 Oct 18;15:48. doi: 10.1038/s41368-023-00253-0 (PMC10584904; doi:10.1038/s41368-023-00253-0)
Supplement: Supplementary file 2 — Supplementary Table 2. The differentially expressed genes in WDR5 overexpressed SCAP compared with Vector group [file 41368_2023_253_MOESM2_ESM.docx]

**Supplementary Table 2. The differentially expressed genes in WDR5 overexpressed SCAP compared with Vector group**

| **Gene Symbol** | **mRNA_Accession** | **Fold Change** | **p-value** | **diffState** |
| --- | --- | --- | --- | --- |
| CYP1B1 | NM_000104 | 2.51 | 0.001349 | up |
| PRSS3 | NM_001197097; NM_001197098; NM_002771; NM_007343; XM_011517965 | 2.49 | 0.000652 | up |
| NPTX1 | NM_002522 | 2.41 | 2.69E-07 | up |
| PYGL | NM_001163940; NM_002863 | 2.36 | 0.000039 | up |
| POSTN | NM_001135934; NM_001135935; NM_001135936; NM_001286665; NM_001286666; NM_001286667; NM_006475; XM_005266231; XM_005266232 | 2.24 | 0.000006 | up |
| HAS3 | NM_001199280; NM_005329; NM_138612; XM_005255921; XM_011523061 | 2.21 | 0.002872 | up |
| TIAM1 | NM_003253; XM_005261037; XM_005261038; XM_005261040; XM_011529711; XM_011529712; XM_011529713 | 2.01 | 0.032405 | up |
| SOBP | NM_018013; XM_005267041; XM_005267042; XM_011535920; XM_011535921; XM_011535922; XM_011535923 | 2.01 | 0.002632 | up |
| APCDD1L | NM_001304787; NM_153360; NR_130908; XM_011528616 | 2 | 0.000348 | up |
| ASNS | NM_001178075; NM_001178076; NM_001178077; NM_001673; NM_133436; NM_183356 | -2 | 0.00003 | down |
| ECM2 | NM_001197295; NM_001197296; NM_001393 | -2 | 0.000013 | down |
| CHAC1 | NM_001142776; NM_024111 | -2.01 | 0.000237 | down |
| NUPR1 | NM_001042483; NM_012385 | -2.02 | 0.000214 | down |
| FBN2 | NM_001999 | -2.02 | 0.013193 | down |
| CBS | NM_000071; NM_001178008; NM_001178009; XM_011529773; XM_011529774; XM_011529775; XM_011529776; XM_011529777; XM_011529778; XM_011529779; XM_011529781; XM_011529782; XM_011529783; XM_011529784; XM_011546094; XM_011546095; XM_011546096; XM_011546097; XM_011546098; XM_011546099; XM_011546100; XM_011546101 | -2.05 | 0.000084 | down |
| NR4A3 | NM_006981; NM_173198; NM_173199; NM_173200; XM_005252237; XM_011519048; XM_011519049 | -2.08 | 0.000035 | down |
| SLC1A4 | NM_001135581; NM_001193493; NM_003038; XM_006712079 | -2.11 | 0.000265 | down |
| SEL1L3 | NM_001297592; NM_001297594; NM_015187; XM_011513818; XM_011513819; XM_011513820 | -2.13 | 0.000609 | down |
| IL6 | NM_000600; XM_005249745; XM_011515390; XM_011515391 | -2.17 | 0.000013 | down |
| VLDLR | NM_001018056; NM_003383; XM_011518029 | -2.41 | 0.000224 | down |
| PSAT1 | NM_021154; NM_058179 | -2.41 | 0.00001 | down |
| G0S2 | NM_015714 | -2.42 | 0.011014 | down |
| EGR1 | NM_001964 | -2.46 | 0.000014 | down |
| FOSB | NM_001114171; NM_006732; XM_005258691 | -2.49 | 0.000003 | down |
| ID4 | NM_001546 | -2.53 | 0.000511 | down |
| LOC102724428 | NM_173354; XM_006723920; XM_006726803; XM_011529474 | -2.62 | 0.000894 | down |
| TAC1 | NM_003182; NM_013996; NM_013997; NM_013998 | -2.71 | 0.001987 | down |
| INHBE | NM_031479 | -2.72 | 0.000344 | down |
| NR4A1 | NM_001202233; NM_001202234; NM_002135; NM_173157; NM_173158; XM_005268822; XM_005268824; XM_006719363; XM_006719364; XM_011538250; XM_011538251 | -2.91 | 0.000009 | down |
| GDF15 | NM_004864 | -3.13 | 0.000189 | down |
| HES1 | NM_005524 | -3.17 | 0.00003 | down |
| ATF3 | NM_001030287; NM_001040619; NM_001206484; NM_001206485; NM_001206486; NM_001206488; NM_001674; NM_004024; XM_005273146; XM_011509579 | -3.59 | 0.000002 | down |
| COL15A1 | NM_001855; XM_011518214 | -3.62 | 0.000105 | down |
| PTGS2 | NM_000963 | -3.68 | 0.000021 | down |
| NR4A2 | NM_006186; NM_173171; NM_173172; NM_173173; XM_005246621; XM_005246622; XM_005246623; XM_006712553; XM_011511246; XR_427087 | -4.6 | 0.003131 | down |
