# Supplementary material for: MLL1 inhibits the neurogenic potential of SCAPs by interacting with WDR5 and repressing HES1
Source: Int J Oral Sci. 2023 Oct 18;15:48. doi: 10.1038/s41368-023-00253-0 (PMC10584904; doi:10.1038/s41368-023-00253-0)
Supplement: Supplementary file 3 — Supplementary Figure Legends [file 41368_2023_253_MOESM3_ESM.docx]

**Supplementary Figure 1. Flow cytometric characterization of SCAPs with cell markers.** CD90 (A), CD105 (B), CD146 (C), CD34(D), CD45 (E).

**Supplementary Figure 2. MLL1 knock-down reduced the expression level of H3K4me3 in SCAPs.** The H3K4me3 level was tested by Western blot. β-actin served as an internal control.
