# Supplementary figures and images for: MLL1 inhibits the neurogenic potential of SCAPs by interacting with WDR5 and repressing HES1
Source: Int J Oral Sci. 2023 Oct 18;15:48. doi: 10.1038/s41368-023-00253-0 (PMC10584904; doi:10.1038/s41368-023-00253-0)

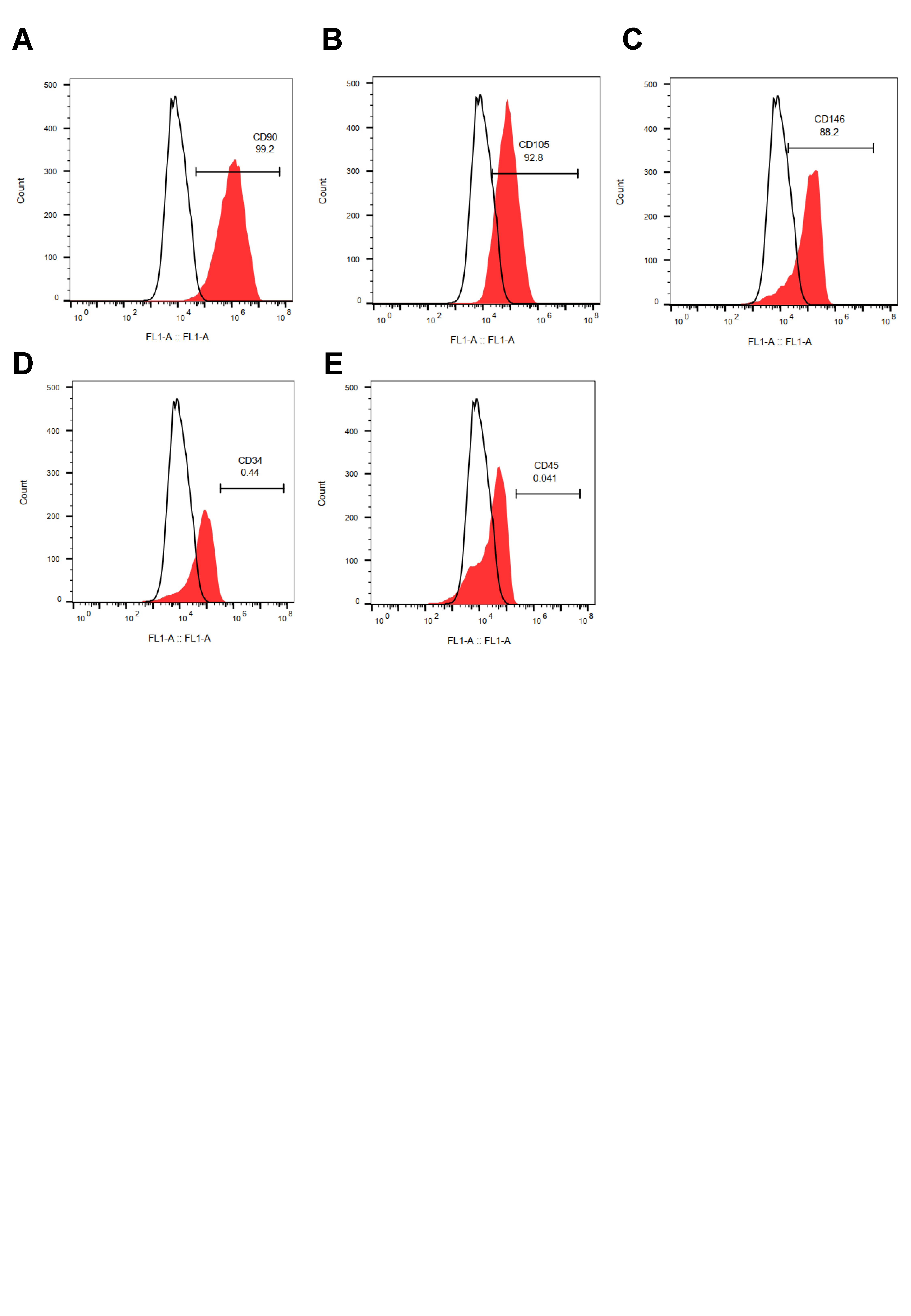

Supplement: Supplementary file 4 — Supplementary Figure 1 [file 41368_2023_253_MOESM4_ESM.tif]

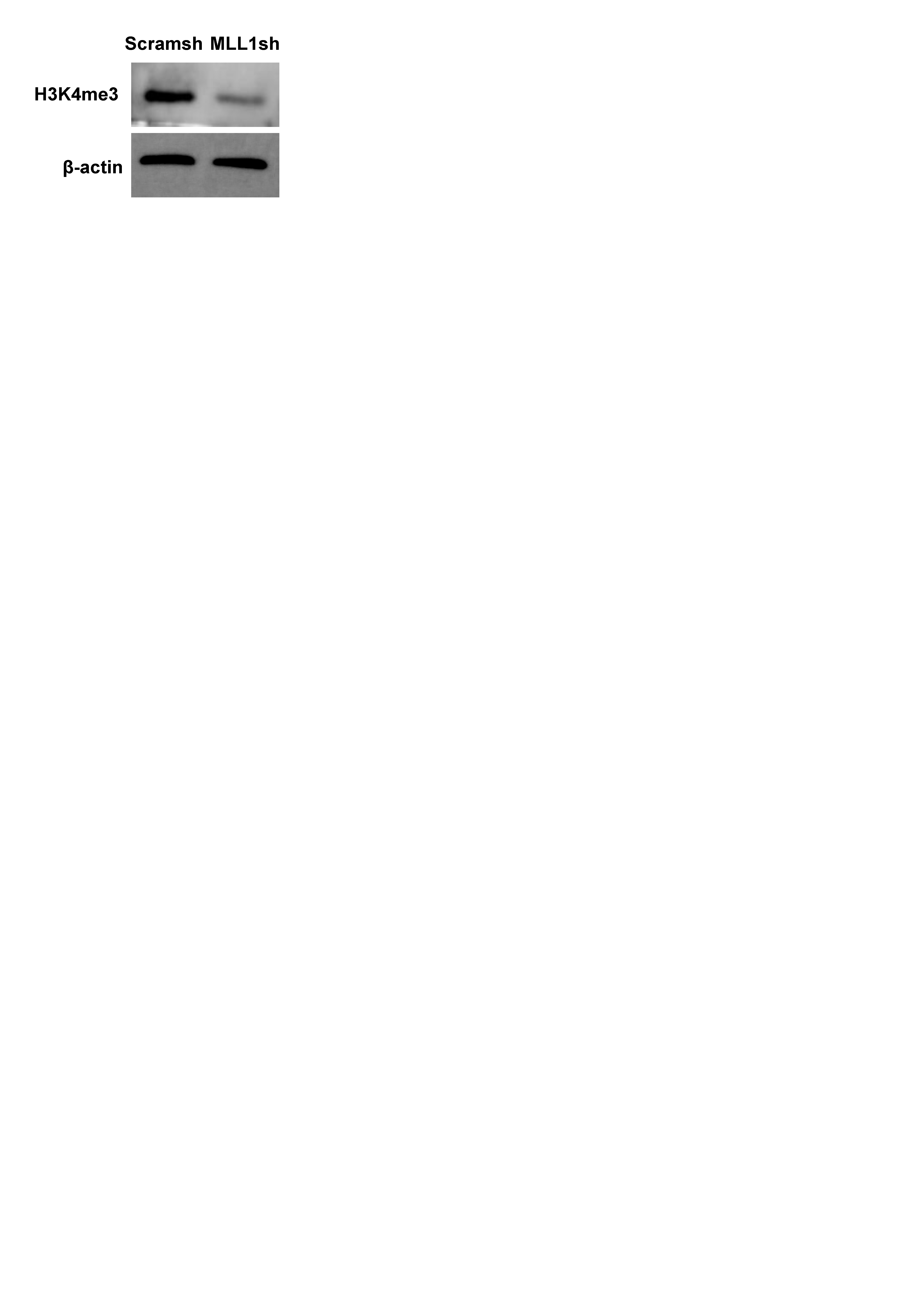

Supplement: Supplementary file 5 — Supplementary Figure 2 [file 41368_2023_253_MOESM5_ESM.tif]
